# The Cost-Effectiveness of 1% Or Less Media Campaigns Promoting Low-Fat Milk Consumption

**Published:** 2005-09-15

**Authors:** Bill Reger-Nash, Margo G Wootan, Steve Booth-Butterfield, Linda Cooper

**Affiliations:** West Virginia University, Department of Community Medicine; Center for Science in the Public Interest, Washington, DC; Department of Community Medicine, West Virginia University, Morgantown, WVa; Department of Community Medicine, West Virginia University, Morgantown, WVa

## Abstract

**Introduction:**

The purpose of our study was to compare the cost-effectiveness of four strategies using components of *1% Or Less *to promote population-based behavior change. *1% Or Less *is a mass-media campaign that encourages switching from high-fat (whole or 2%) to low-fat (1% or skim) milk.

Using a quasi-experimental design, campaigns were previously conducted in four West Virginia communities using different combinations of 1) paid advertising, 2) media relations, and 3) community-based educational activities. Telephone surveys and supermarket milk sales data were used to measure the campaigns' effectiveness.

**Methods:**

Using data from the previously completed studies, we analyzed the cost of each campaign. We then calculated the cost per person exposed to the campaign and cost per person who switched from high- to low-fat milk.

**Results:**

The combination of paid advertising and media relations was the most cost-effective campaign, with a cost of $0.57 per person to elicit a switch from high- to low-fat milk, and the combination of media relations and community-based educational activities was the least cost-effective campaign, with a cost of $11.85 per person to elicit a switch.

**Conclusion:**

Population-based campaigns using a combination of paid advertising and media relations strategies can be a cost-effective way to promote a behavior change in a community.

## Introduction

### Milk and health

Numerous epidemiological studies indicate that diet plays a major role in premature morbidity and mortality in the United States (1). Poor diet and a lack of physical activity may eventually overtake smoking as the leading preventable causes of death (2). Campaigns promoting positive changes in dietary behavior have great potential for improving the public's health.

Milk is a good choice for use in a community campaign to improve health behaviors because it is consumed by so many people and plays an important role in health and the diet. High-fat milk contributes significant amounts of excess calories and saturated fat to the American diet, is the third-leading source of saturated fat in the diet of American adults, and is the leading source of saturated fat in the diets of children aged older than 2 years (3-6). Saturated fats raise blood cholesterol levels and increase the risk of coronary heart disease (1,7). In contrast, skim milk has 40% fewer calories and 5 fewer grams of saturated fat per cup than whole milk. Only six types of food contribute about half of the saturated fat consumed by American adults (4), so we chose one of the items — milk — as the focus of our campaign. (A more recent study found that seven types of food contribute half of the saturated fat consumed by the average American [3].)

### *1% Or Less *campaigns

Mass-media campaigns may be an effective way to address diet-related population health behaviors, and paid mass-media campaigns have been shown to be a useful way to deliver a public health message to numerous people (8). Use of paid television, radio, and newspaper advertising combined with effective media relations have been shown to significantly affect health behaviors when a high level of market penetration is achieved (i.e., when the target audience is repeatedly exposed to the campaign message) (9).

In 1995, The Center for Science in the Public Interest began developing a mass-media community campaign — *1% Or Less* — to encourage one important behavioral change: switching from high-fat (whole or 2%) to low-fat (1% or skim) milk (10) — to encourage one important behavioral change: switching from high-fat (whole or 2%) to low-fat (1% or skim) milk (10). The campaign involved three basic components: 1) paid advertising, 2) media relations, and 3) community-based educational programs. The campaign was implemented in numerous communities nationwide, and the results of the pilot campaigns have been published (11-13).

In this study, we assess the cost-effectiveness of various combinations of the *1% Or Less* campaign components in four individual West Virginia communities. We compare the cost, exposure, and outcomes to the campaign message of the four different types of campaign combinations: 1) paid advertising, media relations, and community-based educational activities; 2) paid advertising and media relations; 3) media relations and community-based educational activities; and 4) paid advertising alone. The paid advertising component consisted of professionally produced, strategically placed television, radio, and newspaper advertising. Media relations comprised events designed and implemented to generate coverage by the local news media. The community-based educational activities included events such as blind milk taste tests in grocery stores, point-of-purchase signs about the program, school activities such as poster-design contests, and nutrition seminars conducted by trained speakers at work sites and for various organizations.

### Background of analyzed studies

The health goal of the campaigns was to encourage community members (older than 2 years of age) to switch from high-fat to low-fat milk. The health communications research goal was to understand how the various combinations of the three *1% Or Less* components work in community-based campaigns. The methodology was approved by the West Virginia University Institutional Review Board.

A quasi-experimental design was used in communities with populations ranging from 18,000 to 35,000. Each of the four communities received an intensive 6- to 8-week campaign, and each campaign involved a different combination of the three *1% Or Less* components (Table 1). The campaigns had a rolling field experiment design. All campaigns were conducted during February and March for 3 consecutive years beginning in 1996 (Table 1). A matched comparison community for each campaign community was observed and received none of the campaign messages. None of the communities had overlapping media markets and thus were not exposed to the other communities' *1% Or Less* campaign messages. All four communities had similar demographics, and their campaigns had similar budgets (11-13).

The Clarksburg, Beckley, and Wheeling campaigns all incorporated the paid advertising component and delivered the *1% Or Less *message to television viewers (and therefore to significantly more people than those actually living in the cities for which data were collected). The television message was delivered to approximately 278,000 (Clarksburg), 363,000 (Beckley), and 418,000 (Wheeling) viewers (11-13). The Parkersburg campaign, which did not incorporate the paid advertising component but had extensive local newspaper coverage, delivered the *1% Or Less* message to approximately 22,500 community members, which is the number of people who subscribed to the local daily newspaper.

The first trial was conducted in Clarksburg and included all three *1% Or Less* components (paid advertising, media relations, and community-based educational activities) (11). The campaign in Wheeling consisted of paid advertising and media relations (12), and the Parkersburg trial involved a combination of media relations and community-based educational activities (13). The community trial in Beckley involved paid advertising only (13). The Parkersburg and Beckley campaigns were conducted simultaneously and were compared with the same comparison community.

In each campaign and comparison community, we conducted random-digit–dial telephone surveys of milk purchasing and consumption habits for approximately 400 adults immediately before the beginning of the campaign (11-13). A panel design was used. We called the baseline respondents again immediately after the campaign and were able to reinterview 69% of the respondents in Clarksburg, 73% in Wheeling, 67% in Parkersburg, and 67% in Beckley. We collected 1 month of fluid milk sales data from all supermarkets in the campaign and comparison communities for the month immediately before and the month immediately after (i.e., beginning the day after), 6 months after, and 12 months after the campaigns ended. In addition, 2 years after the campaign ended in Wheeling, we collected fluid milk sales data for 1 month. Communities were not randomly assigned to campaign or control.

## Methods

Campaign costs (not including evaluation costs for telephone survey and milk sales data collection) were determined by adding the costs of the paid media advertising, personnel salaries, travel, communications, incentives, and meetings. These data provided a total campaign cost per community. The costs per person exposed to the campaign and per person who switched from high- to low-fat milk were determined for each approach based on the telephone survey responses received at the immediate end of the campaigns.

The primary outcome measures for the study were milk consumption survey self-reports and supermarket milk sales from the campaign and comparison communities. We compared precampaign and postcampaign (immediately after the campaign) milk consumption and sales and compared precampaign and 6 months postcampaign milk sales.

For self-reported consumption and milk sales, we computed effect sizes (*r* and *d*), comparing changes in campaign communities with changes in comparison communities (14). Using Cohen's conventions for interpreting effect size (14), we defined a *small* effect as an *r* from 0.1 to 0.2 or a *d* from 0.2 to 0.4; a *medium* effect as an *r* from 0.3 to 0.4 or a *d* from 0.5 to 0.7; and a *large* effect as an *r* of 0.5 or greater or a *d* of 0.8 or greater. However, we did not compute an effect size for the campaign community, compute an effect size for the comparison community, and then perform the analysis. Although our computation is conservative, it allows direct comparisons within each of our campaigns and does not overly weigh extreme results.

## Results

### Changes in low-fat milk sales

The Clarksburg campaign included paid advertising, media relations, and community-based educational activities, which increased low-fat milk sales from 18% to 41% (Table 2). The low-fat milk sales were still higher (33%) 1 year after the campaign ended. Wheeling's campaign involved paid advertising and media relations and increased the low-fat milk sales from 29% to 46%, a change that was sustained at 42% 2 years after the campaign ended. Analysis shows that the increases in low-fat milk sales were statistically significant in the Clarksburg and Wheeling campaigns  (Table 3) (Table 4). Smaller increases in low-fat milk sales were documented after the Parkersburg campaign, which used media relations and community-based educational activities, and after the Beckley campaign, which only used paid advertising. The increases were not significant.

Tables 3 and 4 provide the descriptive statistics for low-fat milk sales in each of the four communities. The treatment effect for the precampaign to postcampaign (the day after the campaign ended) increase in low-fat milk sales (expressed as *r*) ranged from 0.01 (for the paid advertising campaign in Beckley) to 0.64 (for the Clarksburg campaign, which used paid advertising, media relations, and community-based educational activities) (Table 3), with a mean of 0.41. The average *d* effect size was 1.01 (*z* = 2.89, *P* = .002), which is a large effect. Increases in sales of low-fat milk in campaign communities were an average of 1 standard deviation larger than in comparison communities. No statistically significant between-group heterogeneity was found (χ^2^ <1.00); that is, no statistically significant differences were found between campaigns, in spite of the large differences in effect sizes. However, given the wide range of effect size results (0.02 in Beckley to 1.67 in Clarksburg), the lack of statistically significant heterogeneity may be a result of the small sample size.

The precampaign to 6-month postcampaign effect (*r*) for low-fat milk sales ranged from 0.01 (Beckley) to 0.59 (Clarksburg) (Table 4). The average *d* effect was 0.84 (*z* = 2.89, *P* = .002), which is also a large effect. Increases in low-fat milk sales in campaign communities were an average of 80% of a standard deviation larger than in comparison communities. Assessment of between-group heterogeneity revealed no differences (*χ* ^2^ <1.00).

### Self-reported switching to low-fat milk

The treatment effect across the four *1% Or Less* campaigns as measured by survey respondents who reported switching from high- to low-fat milk ranged from 13% (Beckley) to 38% (Clarksburg) (Table 5). In Clarksburg, 38% of respondents who reported consuming whole or 2% milk before the campaign reporting drinking 1% or fat-free milk immediately after the campaign (*P* <.001). In Wheeling, 34% switched from high- to low-fat milk after the campaign (*P* <.001). In Parkersburg, 20% (*P* <.001) switched, and in Beckley, 13% (*P* <.001) switched.

Expressed as an *r* effect, the switching rates range from 0.10 (Beckley) to 0.39 (Wheeling), with a mean of 0.25. The average weighted *d* effect size was 0.53 (*z* = 3.03, *P* <.001), a medium effect (Table 6). These results indicate that self-reported switching in campaign communities was approximately half a standard deviation greater than in the comparison communities. A test for heterogeneity indicated significant heterogeneity between the different campaigns (*χ* ^2^_3_ = 8.606; *P* = .02) measured at the survey sample. Although the average effect was significant, results suggest a discernable difference in the effectiveness of the various campaigns.

### Campaign exposure

Campaign exposure was assessed by asking telephone survey respondents about their awareness of the *1% Or Less* message. The last column of Table 5 shows percentages of self-reported exposure to the campaigns in the four campaigns. We computed a linear contrast among the four effects (weights of 3, 1, −1, and −3), producing a significant effect (*z* = 3.161; *P* = .003). We also explored the possibility of nonlinear effects (weights 1, −1, −1, 1) and found a smaller but still significant effect (*z* = 2.12, *P* = .02).

We used several comparisons to explore patterns of nonlinearity. The strongest pattern suggests that campaigns in Clarksburg and Wheeling resulted in greater changes in low-fat milk consumption than in Parkersburg and Beckley (*z* = 2.72, *P* = .003) (Table 6). No reliable difference between the effects of the Clarksburg and Wheeling campaigns (*z* <1.00) was found, nor was a reliable difference found between the Parkersburg and Beckley results.

### Campaign cost

The cost of each campaign is shown in Table 7. Overall costs of each campaign were similar, ranging from $43,000 (Wheeling) to $61,000 (Clarksburg). In contrast, the number of people exposed to each campaign varied widely. As a result, the approximate cost per person exposed to each campaign ranged from $0.10 (Wheeling) to $2.27 (Parkersburg).

We estimated how much it cost in each campaign to cause one person to switch from high- to low-fat milk. The wide variation in the number of people exposed coupled with differences in switching rates among the campaigns contributed to a wide range in cost. The cost to cause one person to switch from high- to low-fat milk ranged from $0.57 (through the paid advertising and media relations campaign in Wheeling) to $11.85 (through the community-based educational activities and media relations campaign in Parkersburg). The combination of paid advertising and media relations in Wheeling cost approximately $0.10 per person exposed, whereas the Parkersburg campaign (which involved media relations and community-based educational programs) cost $2.27 per person exposed.

## Discussion

All campaigns effectively encouraged people to switch from high- to low-fat milk, but the most cost-effective campaign was the Wheeling campaign combination of paid advertising and media relations. In Wheeling, 34% of high-fat milk drinkers switched to low-fat milk, with a cost of $0.57 per person (Table 7). In addition, statistical analyses show that switching from high- to low-fat milk was not significantly enhanced by the addition of community-based educational programs, and the costs and complexity of the campaigns were greatly reduced when they were not included.

Overall, the results of our analysis of the previous campaigns suggest that the combination of paid advertising and media relations and the combination of paid advertising, media relations, and community-based educational programs are more cost-effective than the combination of media relations and community-based educational programs or paid advertising only. The effect sizes were significantly larger in the two communities that received a campaign combination of paid advertising and media relations. In addition, the two campaigns were more cost-effective, with an estimated cost per person who switched of $0.57 (Wheeling) and $0.73 (Clarksburg), compared with $1.56 for the paid advertising only (Beckley) and $11.85 for the media relations and community-based educational activities combination (Parkersburg) (Table 7). We designed the four campaigns so that they would roughly cost the same amount because the communities were approximately the same size (i.e., were all small, rural cities). Media relations enhanced the impact of paid advertising. The campaign with paid advertising only resulted in approximately 13% of high-fat milk drinkers switching to low-fat milk, compared with 34% in the campaign in which paid advertising was reinforced by media relations (Table 5).

The level at which community members were exposed to the campaign message is a likely contributing factor in the varying effectiveness levels of the four *1% Or Less* campaigns. A linear relationship was found between campaign exposure and the percentages of people switching from high- to low-fat milk. Survey data suggest that some communities had high exposure rates, with 84% in Wheeling and 90% in Clarksburg (Table 5). In other studies, lower exposure campaigns also had less impact (15).

We suggest that health educators change their approach. Although paid media-based campaigns may seem expensive to traditional health educators, our study suggests that paid media-based campaigns are more cost-effective than traditional approaches because of lower personnel and material costs, broader exposure, and greater message reinforcement. Public health organizations may perceive community-based educational programs as more cost-effective because staff costs are already incorporated into their budgets, whereas discretionary funding for advertising is not. However, in the *1% Or Less* campaigns, we found the traditional community-based educational program approach to be the least cost-effective means of switching people to low-fat milk. Furthermore, it is inappropriate to consider overall campaign costs only. The more relevant variable is cost per person who switched.

Several public health publications state that mass media cannot effectively cause population-based health behavior changes. For example, the National Cancer Institute's publication *Making Health Communication Programs Work* (15) and the National Cholesterol Education Program's *Communications Strategy for Public Education* (16) argue that health communications alone cannot produce behavior changes; however, the claims are not directly substantiated by any studies or data. In sharp contrast, the food industry uses mass media as a primary means of influencing food choices, spending about $26 billion per year in advertising and promotions (17).

Evidence is mounting that properly designed mass-media campaigns can produce significant and positive results (8,11-13,18). We subsequently used a media-based approach to promote walking (18). The group exposed to an 8-week media campaign on walking demonstrated a 14% net increase in 30 minutes of moderate-intensity daily walking compared with a control community. The Centers for Disease Control and Prevention's (CDC's) VERB campaign uses paid advertising, public relations activities, and community events to encourage children aged 9 to 13 years to be physically active (19). The results from the first year of this national media campaign show measurable increases in activity levels in key segments of the target audience, including among girls and among children from low-income families (20). Media-based tobacco campaigns have also had positive results (21-23). Effective campaigns that reach broad audiences may be even more cost-effective for promoting nutrition-related behavior change than for decreasing tobacco use because everyone obviously needs to eat, whereas not everyone uses tobacco. Furthermore, the overwhelming majority of Americans (approximately 88%) do not follow federal dietary recommendations (24); perhaps the enormous spending for advertising by the fast-food industry plays a role in this phenomenon. 

Health educators need to join forces with social marketing specialists and sophisticated media production firms to produce high-quality, effective materials. The original *1% Or Less* message was designed by public health, nutrition, marketing, and advertising specialists. In our study, we chose an advertising firm (Zimmerman & Markman, Los Angeles, Calif) to design and produce our television, radio, and print advertisements. After the materials were produced, we worked with a professional media buyer to strategically place the advertisements in a way that would best reach a target audience within the given budget.

Strategic placement of advertisements can result in delivery of a high-impact message and reach the intended target audience. In contrast, health educators using advertisements without professional assistance may spend their time and resources producing public service announcements that result in little market penetration (25). We know of no effective nutrition education campaigns that effectively used public service announcements to produce a significant communitywide behavior change.

Without market penetration (i.e., if campaigns do not reach their intended target), campaigns have little possibility of success. The costly and unsuccessful COMMIT trial failed to promote smoking cessation among heavy smokers — the campaign target (26). Overall, telephone survey respondents reported little knowledge of the COMMIT campaign. (However, in certain communities with more market penetration, the campaign impact was greater.) Although market penetration is a necessary condition for behavioral change, market penetration alone will not result in change.

Our study has implications for primary prevention of heart disease and obesity. Switching from whole to skim milk could result in 5 fewer pounds of fat being consumed by a person in a year. (Because we had individual respondent data from precampaign and postcampaign surveys that were only 3 months apart, we did not anticipate any measurable changes in participants' body weight.)

The generalizability of the *1% Or Less* low-fat milk campaign results is limited by the small number of communities and the lack of random assignment. In addition, the campaigns were conducted at the community level, whereas the telephone survey results were analyzed at the individual level. Our community campaign had survey ecological and survey population measures. No indication shows that a secular trend altered the communities in a way that might have affected the outcomes. The four campaigns and outcome measures spanned 3 years.

Finally, the campaigns were implemented in small-city markets. We are unsure how successful the *1% Or Less* campaign would be in large media markets. We successfully promoted our campaigns as newsworthy and achieved coverage on television news programs and the front page of local newspapers in media markets of 278,000 to 418,000 individuals. Although the campaigns were prominent news items in small media markets, generating news coverage in substantially larger media markets would be more difficult. Additional *1% Or Less* campaigns need to be tested in the media markets of larger metropolitan areas.

Despite these limitations, the data are compelling. The pretest-to-posttest design and measurement of self-reported behavior and communitywide sales provide consistent data with relatively good control (27). The results suggest that dietitians and other health educators should consider the combination of paid advertising and media relations as a central dietary change strategy. The approach might be used as a cost-effective way to promote other dietary changes such as eating more fruits and vegetables or whole grains, switching from butter or stick margarine to lower fat tub margarine, or choosing reduced-fat cheese instead of full-fat cheese.

## Figures and Tables

**Table 1:** *1% Or Less* Components Used in Each West Virginia Community Campaign

**Campaign Community^b^**	***1% Or Less *Components^a^**		

	**Paid Advertising**	**Media Relations**	**Community-based Educational Activities**
Clarksburg (7-week campaign, 1996)	●	●	●
Wheeling (6-week campaign, 1997)	●	●	
Parkersburg (8-week campaign, 1998)		●	●
Beckley (6-week campaign, 1998)	●		

a A bullet (●) indicates that the component was part of the campaign.

b Each campaign took place in February and March for 3 consecutive years beginning in the year indicated.

**Table 2:** Low-Fat Milk Sales Before and After Campaigns

**Campaign Community**	**Strategies Used**	**Low-Fat Milk Sales (Percentage of Total Fluid Milk Sales)^a^**				

		**Precampaign**	**Postcampaign**	**6-Month Follow-up**	**1-Year Follow-up**	**2-Year Follow-up^b^**
Clarksburg	1. Paid advertising 2. Media relations 3. Community-based educational activities	18	41	35	33	NA
Wheeling	1. Paid advertising 2. Media relations	29	46	42	44	42
Parkersburg	1. Media relations 2. Community-based educational activities	28	34	27	27	NA
Beckley	1. Paid advertising	23	28	29	30	NA

a Percentage of total fluid milk sales was determined in the four communities by collecting milk sales data for 1 month before the campaign (precampaign), immediately after the campaign (postcampaign), and 6 months, 1 year, and 2 years after the campaign ended.

b NA indicates not applicable. Supermarkets were reluctant to share milk sales data. Wheeling supermarkets were the only stores willing to provided the requested data after 2 years.

**Table 3:** Descriptive Statistics for Low-Fat Milk Sales Before and Immediately After Campaigns

**Campaign Community**	**Descriptive Statistics**							

	***d***	***r***	***rz***	***N***	***z***	***F***	***df***	***P***
Clarksburg	1.67	0.64	0.76	12	2.75	9.59	1, 12	.003
Wheeling	1.58	0.62	0.73	12	2.23	8.44	1, 12	.013
Parkersburg	0.77	0.36	0.38	12	0.79	1.71	1, 12	.215
Beckley	0.02	0.01	0.01	7	0.01	1.00	1, 7	.965

**Table 4:** Descriptive Statistics for Low-Fat Milk Sales Before and 6 Months After Campaigns

**Campaign Community**	**Descriptive Statistics**							

	***d***	***r***	***rz***	***N***	***z***	***F***	***df***	***P***
Clarksburg	1.46	0.59	0.68	12	2.12	7.66	1,12	.017
Wheeling	1.09	0.48	0.52	12	1.51	4.08	1,12	.066
Parkersburg	0.77	0.36	0.38	12	1.85	5.90	1,12	.032
Beckley	0.02	0.01	0.01	7	0.31	1.00	1,7	.379

**Table 5:** Self-reported Exposure to Campaign and Milk Consumption Before and Immediately After Campaigns

**Campaign Community**	**Strategies Used**	**Percentage of Whole- and 2%-Milk Drinkers Surveyed Who Switched to 1% or Fat-Free Milk**			**Percentage of Surveyed Individuals Exposed to Campaign**

		**Campaign Community**	**Control Community**	***P *Value**	
Clarksburg	Paid advertising, media relations, community-based educational activities	38	10	<.001	90
Wheeling	Paid advertising, media relations	34	4	<.001	84
Parkersburg	Media relations, community-based educational activities	20	7	<.001	71
Beckley	Paid advertising	13	7	.01	50

**Table 6:** Descriptive Statistics for Self-reported Milk Consumption Before and Immediately After Campaigns

**Campaign Community**	**Descriptive Statistics**				

	***d***	***r***	***rz***	***N***	***z***
Clarksburg	0.68	0.32	0.33	136	3.73
Wheeling	0.85	0.39	0.41	147	4.73
Parkersburg	0.38	0.19	0.19	160	2.40
Beckley	0.20	0.10	0.10	155	1.25

**Table 7:** Campaign Cost per Person Exposed and per Person Who Switched From High- to Low-Fat Milk

**Campaign Community**	**Strategies Used**	**Campaign Cost**	**No. People Reached by Campaign**	**Cost per Person Exposed^a^**	**Percentage of Surveyed Individuals Who Switched**	**Approximate No. Who Switched^b^**	**Cost per Person Who Switched^c^**
Clarksburg	Paid advertising, media relations, community-based educational activities	$61,000	278,250	$0.22	30	83,480	$0.73
Wheeling	Paid advertising, media relations	$43,000	418,170	$0.10	18	75,270	$0.57
Parkersburg	Media relations, community-based educational activities	$51,000	22,510	$2.27	13	4,304^b^	$11.85
Beckley	Paid advertising	$51,000	363,050	$0.14	9	32,670	$1.56

a The cost per person exposed to the campaign was calculated by dividing the overall cost of the campaign by the number of people reached by the campaign messages.

b The number of people who switched was based on the population of the campaign community (33,100).

c The cost per person who switched was calculated by dividing the total campaign costs by the total number of people who switched from high- to low-fat milk (determined from the percentage who reported switching).
